# Email Use Reconsidered in Health Professions Education: Viewpoint

**DOI:** 10.2196/19300

**Published:** 2020-06-01

**Authors:** Jennie C De Gagne, Yesol Yang, Sharron Rushton, Paula D Koppel, Katherine Hall

**Affiliations:** 1 Duke University School of Nursing Durham, NC United States; 2 West Virginia University School of Nursing Morgantown, WV United States

**Keywords:** communication, electronic mail, professionalism, faculty, health occupations, health occupations students

## Abstract

Email has become a popular means of communication in the past 40 years, with more than 200 billion emails sent each day worldwide. When used appropriately, email can be an effective and useful form of correspondence, although improper practices, such as email incivility, can present challenges. Email is ubiquitous in education and health care, where it is used for student-to-teacher, provider-to-provider, and patient-to-provider communications, but not all students, faculty members, and health professionals are skilled in its use. This paper examines the challenges and opportunities posed by email communication in health professions education and reveals important deficiencies in training, as well as steps that can be taken by health professions educators to address them. Recommendations are offered to help health professions educators develop approaches for teaching email professionalism.

## Introduction

Given the increasing complexity of the health care system, health professions educators must ensure that future clinicians are prepared to use effective communication intraprofessionally, interprofessionally, and with patients and their caregivers within and across health care settings. Communication skills are foundational competencies in education and patient care [1,2], and health care communication is occurring more frequently in an electronic manner [3]. Although email is ubiquitous in education and health care, its pervasiveness does not ensure that students, faculty members, or health professionals are skillful in its use. In fact, this review of email use within health care and educational settings reveals important training deficiencies and the need for specific steps to be taken by health professions educators. It is imperative that health professionals have the ability to use and select electronic technologies appropriately [1] in order to foster communication and civility among teams in the health care sector.

The birth of email can be traced back to the staff of the Massachusetts Institute of Technology who used electronic notes to communicate on multiuser computers in the 1960s [4]. In 1972, Ray Tomlinson, a computer engineer contracted by the US Defense Department, sent the first electronic message over the earliest form of the internet, the ARPAnet [4]. Ever since email transitioned from technical exchanges among elite programmers to mass communication, researchers have been studying the use of email in higher education institutions [5]. Early studies from the late 1980s focused on the utilization of email as a research tool, user perception and adoption of email in instructional settings, and the effects of email communication on users [5]. In 1997, it was estimated that 17.5 million adults in the United States used the internet for medical information, and by the late 1990s, physicians were beginning to use email for consulting, obtaining laboratory information, following up on patient outcomes, reviewing and disseminating research, and communicating with patients [6]. Today, there are an estimated 5.2 billion registered email accounts globally [7], and they send an estimated 220 billion emails per day [8]. Additionally, 72% of internet users now state that they search the internet for health information [9], and 1%-10% of patients utilize email to communicate with their physicians between appointments [10].

The private, corporate, health care, and higher education sectors incorporate email as a foundational mode of modern communication [11,12]. As such, email has become ubiquitous in higher education and has greatly improved the networking and collaborating capabilities of faculty, staff, and students [13,14]. Email is the means of communication preferred by students and faculty owing to its affordability, accessibility, and ability to send accompanying files [15]. Although the benefits of email include simplicity and speediness of communication, its use can involve unwanted outcomes such as uncivil or inconsiderate behavior. For example, hostile and antagonistic email messages containing aggressive comments, insults, and personal attacks have been frequently reported [16]. Students often take for granted the instant access they have to faculty and take up a considerable amount of faculty members’ work time by asking for information that has been posted [14].

Cyberincivility is defined as communicative behavior against social norms that is exhibited in computer-mediated interactions, such as those involving email and text messages, or on online social networking sites [17]. Because health professional students who demonstrate cyberincivility in school appear to continue the same behavior after they complete their education [18-20], prelicensure education on email netiquette is especially important. In spite of the need, training in email netiquette is not occurring consistently and is not having consistent results in interprofessional discipline training programs. De Gagne et al [16] noted that only half of nursing students reported receiving information on netiquette, with only 6% being aware of the Nursing Council of State Boards of Nursing guidelines on social media. In addition, Oakley et al [20] found that computer-mediated communication guidelines and some training for dental students did not result in adequate outcomes. A study by Barnhart et al [21] reported that the inclusion of training involving the professional use of email in the curriculum for family medicine residents led to some improvements in communication practices, but unwanted behaviors continued.

Given the increased dependence on email in health care and health professions education, and the risk of undesirable outcomes associated with ineffective email communication, it is imperative that health professions educators prepare their students to engage in appropriate and efficient email netiquette. Our viewpoint paper considers the theoretical foundations of netiquette and cybercivility, as well as relevant literature reviews; its purpose is to promote a culture of cybercivility in health professions education in order to foster responsible and effective use of email in the academic and clinical settings.

## Theoretical Foundations of Netiquette and Cybercivility

### Netiquette

Netiquette, or internet etiquette, encourages the use of good manners when communicating in cyberspace [22], thereby promoting users to become better cyber citizens. The core roles of netiquette are to provide ethical and moral concepts of right or wrong, as well as operational guidelines [22] for civil behaviors in the online community. Several theoretical or conceptual frameworks have been posited as the underpinning mechanisms and dynamics behind these social phenomena.

#### Politeness Theory

The politeness theory attempts to explain why people do not always express themselves clearly, directly, or efficiently [23]. According to this theory, people are motivated either by positive faces (ie, a desire to be approved by or connected to others) or negative faces (ie, a desire for disconnection with others or to remain independent) [23]. In order to maintain one’s own positive or negative face, an individual must be socially supportive of others’ needs or faces. When a person feels intimidated by factors, such as disagreement, criticism, disapproval, and skepticism during social interactions, they can respond with a face-threatening act; variations of this protective mechanism include responding (1) without politeness, (2) with positive politeness, (3) with negative politeness, or (4) indirectly or off-record [23]. Although concern has been expressed that the politeness theory may not account for cultural differences in perception or expectations of politeness, it provides an overall foundation for understanding acts of good manners and civility in linguistic and social structures [23,24].

#### Social Information Processing Theory 

The social information processing (SIP) theory, which was coined by Joseph Walther in 1992 [25], explains how people connect and develop relationships in computer-mediated environments without nonverbal signals [26]. Although it is often believed that insufficient verbal cues make it difficult for people to form inferences about others, SIP theorists posit that people can effectively and intimately build a relationship in cyberspace in the absence of face-to-face interactions [26]. In computer-mediated environments where communication is mainly text based (eg, emails, chat rooms, and instant messaging), people can develop favorable impressions of others by seeking out cues in the messages and by choosing words to counteract the lack of nonverbal cues [26]. From SIP perspectives, the characteristics and rate of information exchanged in cyberspace differ from those in face-to-face environments, which may explain why and how uncivil email communications occur and are perceived. The main challenge to the SIP theory is that people often engage in “hybrid” relationships (neither strictly online or offline) [26]. An understanding of how the dynamics of online and offline communication complement each other could advance the development of email civility strategies and other communication techniques to address challenges in cyberspace.

#### Awareness to Action Educational Framework 

In the current digital age, where there is no defining line between public and private space, the private life of a professional can impact their professional image [27]; similarly, an individual cannot separate how they portray themselves in cyberspace from how their character is perceived [27]. The Awareness to Action (A2A) framework encompasses an assessment (proactive) and a decision-making (reactive) tool to facilitate awareness of what is acceptable or unacceptable and appropriate or inappropriate in online communication, and to help individuals make informed decisions regarding online behavior [28]. The three components of the A2A framework (clarity, context, and confirmability, or the three Cs) require an explicit guideline or policy for application to incidents or events [28]. The three Cs should be considered in sequence, and if clarity is not breached, context and confirmability do not need to be assessed [28]. The main question for clarity is as follows: “Does the behavior explicitly breach policy or guidelines?” The question for context is as follows: “Can you explain or describe the context of the situation or when and where it occurred?” and the question for confirmability is as follows: “Can you confirm the consequences and the outcome?” [28]. The A2A framework can be useful for self-efficacy in promoting email civility as it (1) facilitates the reflection of online behaviors and (2) helps to set norms, consensus, consistency, and evidence for decisions in regard to cybercivility.

## Email Use in an Academic Setting

### Academic Cyberincivility

Email provides a number of benefits to faculty and students in the academic setting. Although academics in the late 1980s were reluctant to adopt this new method of communication [29], email has now replaced other modes of communication in higher education [14]. Email is used in traditional and web-based learning environments to facilitate class activities, enable mentoring and collaboration, and disseminate course information and assignments [30-32]. Email is also incorporated into educational environments to facilitate learning and engagement [33]. When used as a pedagogical tool, it allows the instructor to facilitate the dissemination of information and to support conversations that would not normally take place during a class session [13]. Moreover, emails sent by course instructors help to motivate students toward successful learning outcomes [34]. The instantaneous and continuous nature of email permits increased interaction between faculty and students, which is crucial for increasing the quality of education and facilitating an effective learning environment [30]. The asynchronous feature of email supports the careful construction of questions and responses by allowing each party to consider their message before sending [34]. Such a delay can benefit shy or reluctant students by (1) removing the competitive nature of classroom discussions, (2) providing time to reflect on the topic, and (3) allowing students to develop a response that demonstrates a higher level of critical and reflective thinking [34], as well as their communication skills and professionalism [13].

Although there are many positive benefits to email in an academic setting, this form of communication can present challenges related to workload and compromised relationships. Both professionals and academics in higher education are overwhelmed by the number of emails they receive and the pressure to respond to the emails immediately [11]. One study found that associate professors and full professors received an average of 84 emails per working day [14]. When faculty receive overly casual messages from students, they may view the senders as less credible and their messages as poor in quality [13]. Instructors may be less likely to comply with a student’s request after receiving a causal message [13]. Additionally, the relationships between faculty and students and the resulting learning outcomes are at risk of degradation when inappropriate and misinterpreted messages are exchanged [13]. Uncivil emails from students can lead faculty members to have unpleasant feelings toward them and a decreased willingness to collaborate with them [13]; thus, the impact of incivility on relationships is an important rationale for teaching civil behavior in an online environment.

### Email Use Policies in an Academic Setting

Cybercivility is an important component in our increasingly prevalent online interactions and impacts learning in online educational platforms; however, the scope and availability of cybercivility guidelines in US schools of health professions are limited. Email guidelines have been identified for selected schools of health professionals, including a dental school [35] and medical school [36]. A study by De Gagne et al [16] explored the prevalence and composition of cybercivility policies or guidelines regarding email correspondence in US graduate nursing schools (n=230). Only 8% (n=19) of these nursing schools had guidelines for email use. Additionally, best practices for netiquette were found in 84% (n=16) of email guidelines, and 63% (n=12) outlined behaviors to be avoided or reduced. Protocols encouraged a “cooling off” period for emotional and disruptive emails (n=4, 21%) and recommended caution relative to privacy concerns and the potential for miscommunication due to the nonverbal email format (n=9, 47%). Out of 19 guidelines, 9 (47%) specified the consequences for violations. The most common themes found in the guidelines were professionalism, confidentiality/privacy, and forbidden behaviors [16]. These components were also endorsed in previous studies [22,37]. The presence of guidelines for email use can have an impact on the professional and ethical behaviors that are essential for student-faculty relationships [16]; however, guidelines must be accessible and embedded into the curriculum to ensure both awareness and understanding by faculty and students of the professional and ethical behaviors necessary when using email communication [16].

## Email Use in a Clinical Setting

### Intra- and Interprofessional Collaboration

Since the 1990s, health professionals have used email to communicate among colleagues and to schedule meetings [12]. Today, health professionals use email when collaborating and obtaining consults from other professionals because it conveniently enables the dissemination of information, enhances effective communication, and may facilitate patient care [38]. A survey of oncology physicians found that all respondents had used email to communicate with colleagues, including 78% (n=650) who had received results via email [39]. Furthermore, email was found to facilitate communication between inpatient and outpatient settings and was identified as the preferred method of communication among primary care providers [40]. In 2012, a Cochrane systematic review was conducted to determine the effect of email on clinical care. The systematic review identified a single randomized controlled study showing that emails to physicians positively impacted their osteoporosis guideline adherence [41]. In 2015, the authors conducted another systematic review on the same topic but did not identify any new studies [12].

Email communication among providers has been shown to increase the speed and reliability of communication within an interdisciplinary intensive care team, resulting in improved patient outcomes [42]. One study, which looked at the content of emails exchanged between physicians and nurses, found that the majority of emails were of a nonurgent information-sharing nature and that more than 40% did not require any response [43]. In a study of patients with advanced heart failure and ventricular-assist devices, physicians and pharmacists with established connections used email as an adjunct to face-to-face communication for medication management, enabling the initiation and titration of medication therapy [44]. Similarly, a study of smartphone and email use in the clinical setting revealed that the majority of participants felt that email improved their ability to receive a direct and immediate response from other health care providers [45]; however, the study also reported a potential decrease in interprofessional relationships and an increase in uncivil behaviors by trainees who frequently attended to the device [45]. The use of email among professions can entail similar risks. Information inappropriately or incorrectly shared among health care professionals can result in privacy and confidentiality breaches, as well as medical errors [38]. Email incivility in the intraprofessional setting has not been studied extensively, but Resendes et al [37] noted that unprofessional email communication among health providers can induce negative perceptions of the sender and a delay in response time.

### Patient Email Use

Email communication provides a valuable tool for provider-patient interactions when used appropriately and in a secure manner. In fact, many patients prefer communicating with their health care provider via email [20] because it expands opportunities for consultation, treatment, and patient care [38]. Email has been described as environmentally and economically friendly, as well as efficient because it quickly connects the individuals providing and receiving care [46]. In a previous report, 80% of oncology physicians surveyed had communicated with a patient via email [39]. The two most common topics of emails identified by primary care physicians were answering patient questions (82% of respondents; n=219) and changing appointments (72% of respondents; n=192) [47]. Additionally, the use of email can improve the management of chronic diseases and continuity of care because it enables patients to disclose sensitive or embarrassing issues that they might have difficulty discussing face-to-face [48]. Research on the impact of physician email communication has generally been positive with both patients and providers noting convenience and improved quality of care [49-51], although at least one study [52] found that patients preferred telephone or direct communication over email on their military health secure messaging system. In 2018, Wagg et al [53] reviewed 31 studies involving computer-mediated communication (eight of these studies focused on email) and found that 81% (n=26) demonstrated a positive impact on patients. The outcomes noted in this review were increases in access to health care providers, enhancements in communication between patients and providers, improvements in meeting the informational needs of patients, increases in patient empowerment, and improvements in blood sugar control among diabetic patients who received supportive emails. Although the benefits have been noted, physician use of email communication with patients is low compared to both patient and provider willingness (6%-19% vs 70%) [54].

The impact of email on patient-provider communication has been generally positive, but there are concerns about its use. Patients have expressed concerns about whether physicians actually receive their emails, and if so, how quickly [49]. Additionally, socioeconomic indicators of patients have been identified as barriers to email use [49,51]. Makarem and Antoun [55] described much lower use of email communication between patients and physicians in developing countries, as well as differences in how patients and physicians view the use of email and its importance. Physicians using email identified workload, lack of reimbursement for time responding to emails, and inappropriate use of email by some patients as barriers to its effective role [49-51].

The ease and speed of email communication can result in unprofessional and miscommunicated messages [46]. The negligent construction of email messages (eg, no subject lines, no proper salutations, excessive lingo usage, and slang) can negatively impact the professional rapport physicians must maintain with their patients [38]. Missing or inappropriate information provided by patients may cause confusion or a delay in treatment [48]. Furthermore, research demonstrates that the content and tone of emails between patients and providers is generally task-oriented and focuses on nonurgent health-related issues, although some content relates to emotional needs and relationship building [49,56]. While Hogan et al [56] found tone and content to be generally positive, the need for patient-centered improvements and proactive communication by providers was noted. Patients and physicians have shared concerns about confidentiality and security [49-51]; however, Mold et al [51] found no harm or privacy violations in a systematic review of 17 studies.

### Organizational Use of Email

Email has the potential to impact several aspects of organizations. First, health care organizations use email to conduct routine business tasks and value it for its ability to easily share documents, facilitate collaboration and workflow, and hold workers accountable [57]. Second, email facilitates organization information sharing both intraorganizationally and extraorganizationally. Within the organization, email has been used to disseminate evidence-based practice information [58]. A case study by Medland [59] demonstrated that email can be used as a leadership tool to promote connection, build competency, and increase coworkers’ sense of being valued. Outside the organization, the Veteran Administration conducted a survey to better understand how veterans would like to receive information in the event of a natural disaster, and veterans ranked email messages as one of the top three helpful communication modes for those less than 64 years old [60]. Third, email has been utilized by organizations as part of quality improvement efforts. An organization embedded email reminders in electronic health records to improve admission medication reconciliation by resident physicians [61]. Email consults based on a template have also been implemented for professionals ranging from primary care providers to specialists within a national health system, showing a decrease in wait times and reduced cost [62]. Fourth, the use of email has been studied as a means of improving processes from a research perspective. Another hospital developed an automated system to send emails to physicians for tests pending at patient discharge to improve follow-up care [63].

### Email Policies in Health Care

While professional standards have been established for professional communication in the health care sector [46], as well as behavioral health provider standards or guidelines [64], lack of training or guidelines on electronic or email communication for health care professionals has been clearly documented [16,20,46]. It has been suggested that an understanding of how to use email does not necessarily ensure appropriate professional communication [20,65,66]. Accordingly, studies on email communication across health care disciplines, including nursing [16,17], medicine [27,37,38,46,67,68], and mental health [69], have called for the development of policies and guidelines to enhance this form of communication. Malka et al [38] and Railey et al [46] found a lack of formal guidelines for email use by physicians. Guidelines for patient-physician email and text message exchanges have been recently published by the American Medical Association [70] to address such issues, including (1) establishing a turnaround time for messages, (2) retaining copies of email communications with patients, and (3) refraining from sending angry, sarcastic, harshly critical, and libelous references to third parties [70]. In addition to professional organizations, some health care systems have created policies to guide electronic communication among their employees [71,72].

## Recommendations

### Evidence-Based Educational Strategies

We concur with the interventional strategies for integrating cybercivility into the following areas of health professions education proposed by De Gagne et al [73]: (1) ethical knowledge and skills, (2) curriculum development and content delivery, and (3) praxis. Their study demonstrated that students in health professions lack knowledge on e-professionalism and would benefit from online resources that facilitate reflective discussions. They recommend the following: (1) integrating cybercivility and digital communication into course curriculum to facilitate formal assessment and evaluation of these learning objectives; (2) evaluating content to ensure it is accessible, feasible, and effective; (3) incorporating writing and reflective exercises to help uncover and make visible any unwritten or unintended “hidden curriculum;” (4) providing training at both individual and organizational levels; and (5) promoting partnerships and faculty development through cybercivility training, including interprofessional training for currently employed health care professionals [56].

### Existing Models

Faculty can use models that have already been developed in their teaching procedures. Railey et al [46] created the SURE model with key questions for health care providers and students to use when composing an email. In this model, S is related to checking spelling and syntax, and including a subject and signature; U is related to urgency and an unprofessional tone; R is related to reviewing for content and confirming a recipient; and E is related to emotions and ethical concerns [46]. The previously described A2A framework by Ryan [28] is another potential model. Regardless of the models or teaching approaches employed, curriculum for both professional development and health professions education should include the content outlined in the next four subsections of this paper.

#### Basic Email Etiquette

Poorly constructed or uncivil email communications, including those that leave out relevant information (eg, subject line or name) [14], disregard spelling and grammar errors, insert inappropriate abbreviations and slang, or use informal and impolite tones [13,74], can damage the credibility of the sender and cause the reader to underestimate the sender’s competency [13]. While working to understand the unfavorability of selected characteristics in professional emails, researchers identified nonwhite background color, hard to read fonts, and lack of a subheading as the top three unfavorable characteristics that make recipients less likely to reply [37]. Using the “reply all” button when it is not absolutely necessary can also create frustration for those to whom the topic does not apply. Some examples of statements that should never be sent as “reply all” are as follows: (1) Congrats! (2) Thank you! (3) I agree, (4) Please remove me from this mailing list, (5) LOL, and (6) Please stop reply all to this thread [75].

#### Ethical, Legal, and Professional Implications

The importance of writing emails that reflect and communicate professional and ethical values must be emphasized in educational curriculum and practice guidelines. The effects of inappropriate communication on student-faculty relationships, patient-provider relationships, and the reputation of professional sectors within the larger global community are far reaching. Faculty, staff, and students should be aware of how email communication is impacted by federal, state, and local laws; in particular, students and clinicians should be aware of what types of patient information must be excluded from emails in order to comply with the Health Insurance Portability and Accountability Act. Faculty should carefully consider any potential violations of the Family Educational Rights and Privacy Act when communicating with and about students via email.

#### Clear Guidelines for Expectations and Repercussions for Infractions

Cain and Romanelli [27] recommended honor codes and professional socialization as potential techniques to improve professional email communication. Formal guidelines should include clear direction on e-professionalism, cybercivility, and cyberincivility, as well as specific behaviors to be avoided, expectations for conduct, and consequences for inappropriate communication [16]. It is important to define and address the consequences of uncivil behaviors and to provide educational resources for both those enacting uncivil behaviors and their victims [73]. The development of a common set of guidelines and standards across disciplines could be an important collaborative opportunity pioneered by interprofessional health educators.

#### Research

Additional research is needed to provide an increased understanding of email incivility in the clinical and academic settings. Current faculty and professional development approaches for educating students and other professionals on email netiquette should be evaluated in order to identify strategies that enhance learning, encourage behavioral change, and enforce guidelines. Further research is needed to explore the use of email in interprofessional communication, as well as the development of conceptual models, according to the theories discussed in this paper, to support curriculum inclusion and guideline development [23,25]. Finally, additional studies are needed to (1) understand the effective use of email communication between health care providers and patients and (2) identify the types of information and interactions that are most effective for specific populations.

## Conclusions

There is agreement in the literature that email incivility has a negative impact on student-faculty, faculty-faculty, and patient-provider relationships; however, interventions to address this problem in health professions education have not been well documented. This paper discussed current knowledge from a review of the literature on theoretical foundations of cybercivility and made recommendations for strengthening email netiquette. Fostering e-professionalism requires the cultivation of not only knowledge and skill but also ethical and moral reasoning. Given the importance of web-based learning platforms and digital communication, the need for effective strategies for educators is paramount.

## Abbreviations

A2A: Awareness to Action
SIP: social information processing
